# Methods and Assessment Tools for Therapeutic Assignment in Psychotherapy: A Systematic Review

**DOI:** 10.7759/cureus.92110

**Published:** 2025-09-12

**Authors:** Maria Laura Mele, Roberta Meloni, Stefano Federici

**Affiliations:** 1 Center for Research and Psychotherapy, Myèsis Insight Center, Rome, ITA; 2 Department of Philosophy, Social and Human Sciences and Education, University of Perugia, Perugia, ITA

**Keywords:** personalized psychotherapy, systematic review, therapeutic assignment, therapy matching, treatment decision-making in psychotherapy

## Abstract

Psychotherapy has evolved from rigid frameworks to personalized approaches, recognizing that no single method works for all. This has increased focus on “therapeutic assignment,” which matches clients to specific therapies based on individual traits rather than diagnoses. Traditional selections often depend on therapist preference, while diagnoses alone poorly predict treatment response. This highlights the need for research-driven matching based on psychological, situational, cultural, and personal factors. In this systematic review, we analyzed existing methods, models, and assessment tools used to align clients with psychotherapeutic approaches, emphasizing the identification of evidence-based strategies for personalized therapeutic assignment. Empirical evidence increasingly supports the necessity of tailored treatment planning grounded in multidimensional client profiling, rather than relying on traditional models that depend on theoretical orientation or therapist selection. This systematic review aimed to consolidate the current state of knowledge regarding therapeutic assignment in psychotherapy by examining three primary research questions: (i) What methods and assessment tools are utilized to align clients with psychotherapy? (ii) How are client profiles integrated into decision-making models for selecting appropriate treatment options? (iii) What client variables are considered in psychotherapy assignments? In February 2025, a thorough search of the literature was done across several databases, with Scopus being the main one. PsycINFO, PubMed, and Google Scholar were also searched. The search strategy used was systematic and combined terms related to personalized psychotherapy, therapy matching, systematic treatment selection, client characteristics, and treatment decision-making. The search included publications from January 2010 to June 2025, including foundational theoretical works. After reviewing 493 initially identified articles and applying rigorous inclusion and exclusion criteria, 47 eligible studies were included in the qualitative synthesis. The findings highlight essential variables for therapeutic matching, including coping style, motivation, attachment style, and cultural background, while identifying key assessment tools such as the Minnesota Multiphasic Personality Inventory (MMPI), Systematic Treatment Selection Clinician Rating Form (STS-CRF), and different personality and symptom measures. Decision-making models like systematic treatment selection, prescriptive psychotherapy, and feedback-informed approaches provide structured frameworks for therapeutic assignment, with Integrative and modular approaches demonstrating improved adaptability to individualized treatment needs. However, most models stem from Western-centric clinical research, potentially restricting their applicability in culturally and systemically diverse contexts, and the reviewed studies demonstrate limitations in generalizability, as most findings stemmed from disorder-specific applications and empirically validated treatments. The identified limitations highlight the need for an increased emphasis on sociocultural and contextual factors in therapeutic matching research and broader transdiagnostic personalization frameworks that focus on common psychological processes and mechanisms across different diagnostic categories rather than disorder-specific interventions.

## Introduction and background

Psychotherapy has experienced considerable systematic development and refinement in recent decades, transitioning from strict theoretical frameworks to practices that consider individual needs [1]. This evidence-based evolution indicates an increasing acknowledgment that no singular therapeutic method is universally effective [2]. The notion of “therapeutic assignment,” referring to the alignment of individuals with particular psychotherapeutic methods, has become a significant focus in clinical practice [3]. Additionally, integrative approaches combining multiple therapeutic models or methods have emerged as promising frameworks for personalized treatment. However, their systematic evaluation is limited in the literature due to the complexity of configuring and measuring diverse integrated models across different pathological frameworks [4]. Clinicians encounter the challenge of determining the most suitable therapeutic approach for each client, a process that transcends mere diagnostic categorization [5].

Historically, treatment selection has frequently depended on clinician preference or theoretical allegiance, with therapists employing familiar methods regardless of client-specific factors [6]. Studies have indicated that diagnostic categories alone offer limited assistance in personalizing treatment or predicting therapeutic responsiveness [3-6]. This insight aligns with broader trends in healthcare, including personalized medicine, which focuses on customizing interventions according to individual profiles [7]. Psychotherapy is increasingly viewed in a comparable manner, recognizing that clients vary in psychological composition, life circumstances, cultural backgrounds, and individual preferences [8,9].

Despite significant progress in psychotherapy research and practice, a key challenge remains: How can clients be systematically and effectively matched with the most suitable treatment modality? Although no psychotherapeutic approach has been shown to be significantly more effective across different populations [2], individual clients respond differently to specific interventions based on their unique characteristics, preferences, and circumstances [3]. This variability in treatment response underscores the critical importance of personalized matching to optimize outcomes for individual clients rather than relying on population-level effectiveness data. The issue goes beyond mere effectiveness, encompassing matching client expectations and treating psychotherapy as a personalized service with different methods. The complexity of this question is heightened by the numerous psychotherapeutic approaches, each characterized by unique theoretical foundations and mechanisms of change; however, no single approach surpasses others across diverse populations and conditions [3]. The absence of evidence-based matching guidelines may result in suboptimal outcomes, inefficient resource utilization, and decreased adherence, especially when treatments do not align with client characteristics or expectations [10,11].

In this systematic review, we aimed at consolidating the state-of-the-art regarding therapeutic assignment in psychotherapy, focusing on three primary questions: (i) What methods and assessment tools are utilized to align clients with psychotherapy? (ii) How are client profiles integrated into decision-making models for selecting appropriate treatment options? (iii) What client variables are considered in psychotherapy assignments? Therapeutic assignments typically occur during initial assessments, treatment planning sessions, and mid-treatment adjustments. These assignments are performed by various professionals, including therapists, treatment teams, and assessment specialists [3]. In real-world practice, when a therapist’s approach is insufficient for a patient, current patterns often diverge from evidence-based assignment procedures. This highlights the need for systematic protocols for modifying or reassessing treatment approaches [5]. Our results ideally help clinicians, researchers, and policymakers in comprehending, validating, and implementing personalized therapeutic matching practices through the examination of these areas. In the following sections, we outline our methodological approach, present results categorized by research question, and provide critical analysis along with suggestions for future research.

## Review

Method

A comprehensive literature search was conducted in February 2025 for this systematic review. The search spanned multiple databases, with Scopus serving as the primary source given its scope as the largest repository of peer-reviewed literature. Supplementary searches were performed in PsycINFO, PubMed, and Google Scholar to ensure broad coverage. The search spanned publications from January 2010 to June 2025, with the inclusion of seminal works foundational to therapeutic assignment theory (e.g., Larry E. Beutler’s early work [5] on systematic treatment selection).

The research question was structured by using the *population*, *intervention*, *comparison*, and *outcome *(PICO) framework [12]. Keywords were systematically identified by the authors (M.L.M., R.M., and S.F.) through a multi-step process: (i) Initial terms were brainstormed for each PICO component based on the research objectives; (ii) controlled vocabulary terms were identified by searching database thesauri with the initial terms (MeSH headings in PubMed, Emtree terms in Embase, APA Thesaurus terms in PsycINFO); (iii) free-text terms were derived from key papers, serving as primary search terms for Scopus and Google Scholar and as supplementary terms for thesaurus-based databases; (iv) synonyms and alternative spellings were identified with database suggestion tools and relevant literature; and (v) all terms were combined with the use of Boolean operators to create comprehensive search strings adapted for each database. The text string used was: (“personalized psychotherapy” OR “individualized psychotherapy” OR “tailored psychotherapy” OR “adaptive psychotherapy” OR “client-centered psychotherapy”) AND (“therapy matching” OR “systematic treatment selection” OR “psychotherapy customization” OR “context-sensitive psychotherapy”) AND (“client characteristics” OR “individual differences in psychotherapy response” OR “psychological profiling in psychotherapy”) AND (“treatment decision-making in psychotherapy” OR “best-fit psychotherapeutic approach” OR “tailoring psychotherapy to patient needs”) AND (“psychotherapy models” OR “theoretical orientations in psychotherapy” OR “integrative psychotherapy” OR “comparative psychotherapy studies” OR “eclectic therapy”), entered in the generic field of the databases (PubMed, Scopus, EBSCOhost-all databases) without further specification regarding the nature of the study or the population sample investigated.

The inclusion and exclusion criteria are detailed in Table 1.

**Table 1:** Inclusion and Exclusion Criteria

Inclusion criteria	Exclusion criteria
Peer-reviewed articles published in English	Studies not involving human subjects
Studies focused on adult populations (aged 18 years and above)	Non-peer-reviewed articles, including dissertations, theses, and unpublished manuscripts
Research examining therapeutic assignment models, psychotherapy triage, therapeutic assignment frameworks, or clinical decision-making tools	Research focused exclusively on pharmacological treatments without a psychotherapy component
Studies comparing different models of therapeutic assignment, manualized versus personalized therapy, or clinician judgment versus algorithmic matching	Studies involving non-clinical populations (e.g., those focusing solely on self-help, coaching, or workplace interventions without a clinical psychotherapy component)
Research assessing treatment efficacy, dropout rates, therapeutic alliance, or symptom reduction as a function of therapy assignment	Single-case studies or anecdotal reports, unless they contributed to a meta-analysis or systematic review
Publication types including empirical studies, systematic reviews, meta-analyses, and theoretical papers with substantial empirical support	Papers without full-text access
Studies published from 2010 onwards to ensure relevance to current clinical practice while maintaining a comprehensive timeframe for systematic review, with inclusion of foundational theoretical works regardless of publication date	Studies published before 2010, unless they provided foundational theories essential to understanding the field

This study focused on peer-reviewed empirical studies, theoretical papers, and review articles addressing therapeutic assignments in psychotherapy. Case reports, editorials, and non-peer-reviewed publications were excluded, as were gray literature such as conference abstracts and unpublished sources. Backward and forward citation chasing was not conducted. Only publications in English were included, and no other language restrictions were applied.

**Figure 1:**
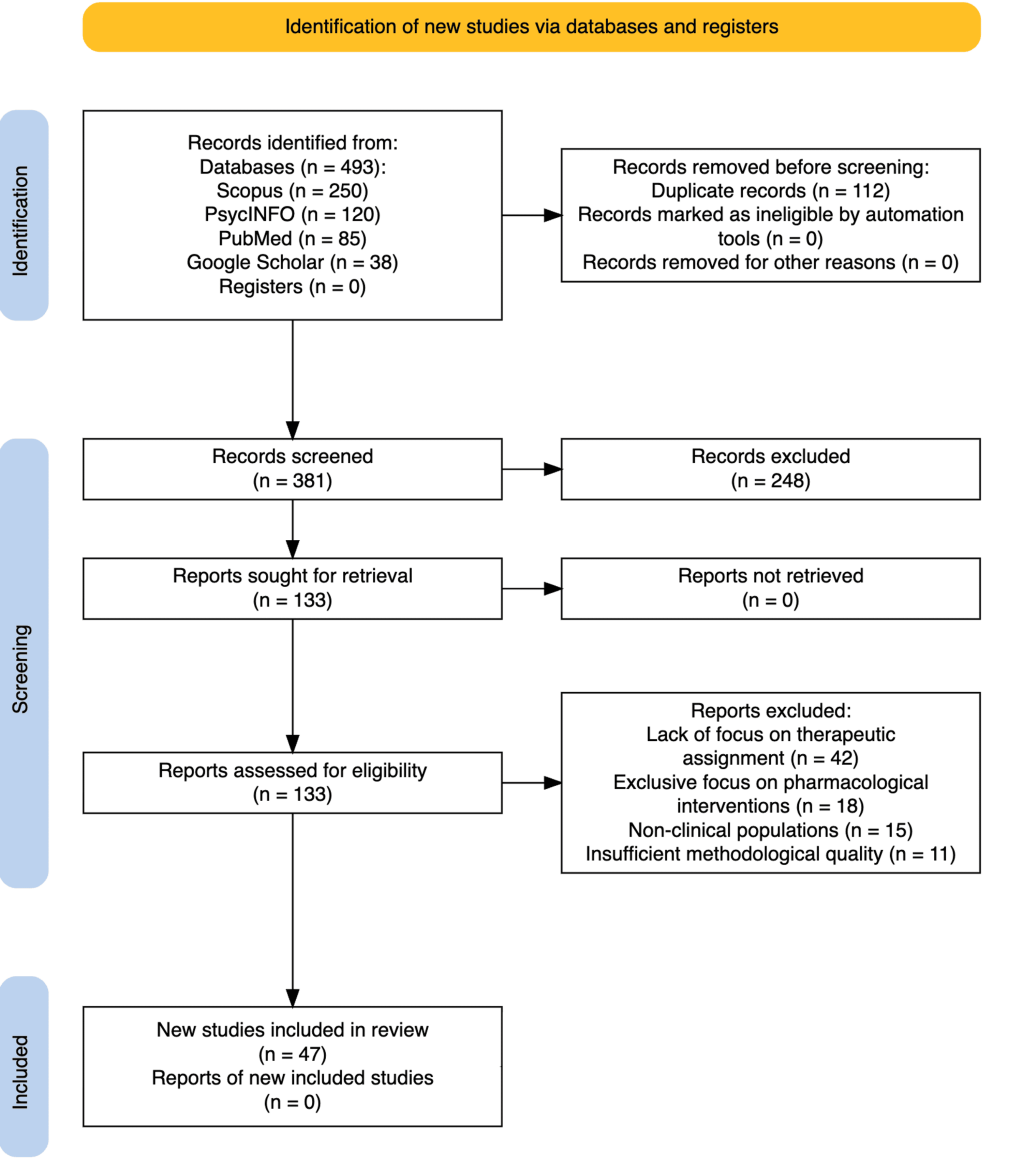
PRISMA 2020 Flow Diagram for New Systematic Reviews. PRISMA: [12]

Our initial search identified 493 articles. After removing duplicates (*n* = 112), 381 unique records remained. Three independent reviewers (M.L.M., R.M., and S.F.) conducted a blind screening of titles and abstracts, using structured consensus procedures to resolve disagreements. Each reviewer assessed all titles and abstracts independently using standardized criteria. Disagreements were resolved through discussion and consensus meetings. This multi-reviewer approach enhanced the reliability of study inclusion decisions and reduced selection bias by ensuring a systematic evaluation process and minimizing individual reviewer bias. Full-text reviews were conducted on 133 remaining articles, with further exclusions due to the lack of focus on therapeutic assignment (*n* = 42), exclusive focus on pharmacological interventions (*n* = 18), non-clinical populations (*n* = 15), and insufficient methodological quality (*n* = 11). The final sample comprised 47 studies included in the qualitative synthesis (Figure 1).

A data extraction form captured the following key variables: author(s), year, study design, sample characteristics, assessment tools, profiling approaches, decision-making models, theoretical orientations, findings, and limitations. Three reviewers (M.L.M., R.M., and S.F.) independently extracted data; discrepancies were resolved by consensus. Results were compiled into an evidence table to support cross-study comparison. Given the heterogeneity of study designs and outcomes, a narrative synthesis was conducted and structured around the main research questions listed in the Introduction.

Results

Next, we present the principal findings of the systematic review structured around the three guiding research questions (RQs): (RQ1) What methods and assessment tools are utilized to align clients with psychotherapy? (RQ2) How are client profiles integrated into decision-making models for selecting appropriate treatment options? (RQ3) What client variables are considered in psychotherapy assignment? The results are summarized in Table 2.

**Table 2:** Methods and Assessment Tools Identified Across 47 Studies (2010–2025) That Examined Therapeutic Assignment in Psychotherapy. Note: Foundational tools and frameworks developed before 2010 are included as seminal works essential to the field and actively referenced in contemporary studies within the review period.

Research Question	Method/Assessment Tool	Authors	Year
RQ1: What methods and assessment tools are utilized to align clients with psychotherapy?	Minnesota Multiphasic Personality Inventory (MMPI)	Hathaway & McKinley [13]	1943
	MMPI-2	Butcher & Williams [14]	2009
	Millon Clinical Multiaxial Inventory-III (MCMI-III)	Millon & Davis [15]	1997
	Vocational Preference Inventory (VPI)	Holland [16]	1985
	Therapeutic Reactance Scale (TRS)	Dowd et al. [17]	1991
	STS-Clinician Rating Form (STS-CRF)	Corbella et al. [18]	2003
		Fisher et al. [19]	1999
	Therapy Process Rating Scale (TPRS)	Ulberg et al. [20]	2016
	Adult Attachment Interview (AAI)	George & West [21]	2011
	Beck Depression Inventory-II (BDI-II)	Dozois [22]	2010
	Symptom Checklist-90-R (SCL-90-R)	Derogatis & Unger [23]	2010
	State-Trait Anxiety Inventory (STAI)	Spielberger et al. [24]	1983
	Dysfunctional Attitude Scale (DAS)	Power et al. [25]	1994
	BASIC I.D. profile	Lazarus [26]	2005
	Systematic treatment selection (STS) framework	Beutler et al. [5]	2005
RQ2: How are client profiles integrated into decision-making models?	Systematic treatment selection (STS)	Beutler et al. [5]	2005
		Consoli & Beutler [27]	2019
	Prescriptive psychotherapy	Groth‐Marnat et al. [28]	2001
		Beutler [29]	2011
	Machine learning approaches	Constantino [30]	2024
		Delgadillo & Gonzalez Salas Duhne [31]	2020
	Bayesian modeling	Zhou et al. [32]	2022
	Ecological momentary assessment	Constantino [30]	2024
	Transtheoretical stages of change	Prochaska & DiClemente [33]	1984
	Adaptive counseling and therapy (ACT)	Howard et al. [34]	1986
	Feedback-informed care	Constantino [30]	2024
		Zilcha-Mano & Fisher [35]	2022
	Millon’s evolutionary model	Millon & Grossman [8]	2007
		Strack & Millon [36]	2013
	Selective and adaptive indication models	Castonguay & Beutler [2]	2005
		Delgadillo & Gonzalez Salas Duhne [31]	2020
		Nye et al. [7]	2023
	Mechanism-focused profiling	Schiepek & Pincus [37]	2023
		Solomonov & Barber [38]	2022
	Modular therapies	Zarbo et al. [39]	2016
	Algorithm-based modular psychotherapy	Schramm et al. [40]	2024
RQ3: What client variables are considered in psychotherapy assignment?	Coping style (internalizing/externalizing)	Beutler et al. [5]	2005
		Brintzinger et al. [41]	2021
	Resistance levels	Beutler et al. [5]	2005
		Lazarus [26]	2005
	Psychological mindedness	Groth‐Marnat et al. [28]	2001
	Motivation and readiness for change	Prochaska & DiClemente [33]	1984
	Functional impairment and symptom severity	Beutler et al. [5]	2005
	Attachment style	Beutler et al. [3]	2018
		Zilcha-Mano & Fisher [35]	2022
	Personality traits (Millon framework)	Millon & Grossman [8]	2007
	Cultural background and expectations	Eggenberger et al. [42]	2023
	Client preferences and beliefs	Philips et al. [43]	2007
		Zuber [44]	2000
	Therapist–client compatibility	Constantino [30]	2024
		Eggenberger et al. [42]	2023

The systematic review identified 47 references, including empirical studies and theoretical contributions, related to therapeutic assignment. The 13 empirical studies included randomized controlled trials (*n* = 3) [31,40,45], observational studies (*n* = 5) [41,43-46], meta-analyses (*n* = 2) [3,7], and instrument validation studies (*n* = 3) [18,20,25]. The remaining references (*n* = 34) included theoretical frameworks (*n* = 17) [1,2,4-6,8-11,26,27,29,33,34,36,37,47], assessment instrument descriptions (*n* = 10) [13-17,19,21-24], and reviews and commentaries (*n* = 7) [28,30,35,38,39,42,48]. Empirical studies focused primarily on clinical populations with depression and mood disorders (*n* = 6) [3,25,31,40,45,46], mixed clinical populations (*n* = 4) [7,18,20,32], and psychotherapy patients (*n* = 3) [41,43,44]. The outcome measures in the empirical studies included treatment response and efficacy (*n* = 5) [3,7,31,40,45], outcome measures and progress indicators (*n* = 3) [32,41,46], and psychometric validation (*n* = 3) [18,20,25]. The geographic distribution of the empirical studies showed a predominance of Europe (*n* = 7), followed by North America (*n* = 3), Asia (*n* = 2), and international collaborations (*n* = 1). This limits the cross-cultural generalizability of the findings.

RQ1: What Methods and Assessment Tools are Used to Align Clients With Psychotherapy?

Therapeutic assignment is transitioning from theory driven, intuition-based methods to evidence-based strategies rooted in individualized profiling [1,44]. Historically, diagnosis-based matching has dominated; however, it demonstrates limited efficacy for individualized planning [5]. A critical limitation identified in the current literature is that many assessment tools were not initially designed for therapeutic assignment decisions, yet they have been adapted for this purpose. This adaptation gap underscores the necessity of specialized assessment instruments for therapeutic matching and reveals the shortcomings of using general assessment tools for these decisions. While the identified tools provide useful data for decision-making, their indirect application to therapeutic assignment may compromise precision and effectiveness. Categorical diagnoses frequently lack predictive specificity and do not correspond with mechanisms of change [5,10]. Consequently, numerous researchers support idiographic and multidimensional frameworks that emphasize client-specific variables [44].

Research has emphasized the significance of interpersonal compatibility and alignment in therapeutic expectations and relational style, indicating that such congruence may improve alliance and decrease dropout rates [11,30,42]. The systematic treatment selection (STS) framework [5] is a formal model that offers a structured methodology, incorporating empirically validated dimensions to enhance decision-making processes. This framework informs the selection of therapeutic modalities by considering various client characteristics such as resistance (associated with negative outcomes in directive approaches), motivational stage, and attachment style [3,27,37].

Emotional processing and therapy preferences are crucial factors in the matching process. Individuals who exhibit externalizing tendencies may derive benefit from structured techniques, whereas those with reflective dispositions may find exploratory interventions more beneficial [47]. Preferences can be articulated directly or deduced from clinical interviews and have been integrated into STS and several decision-making frameworks [46].

Assessment instruments facilitate these processes. The Minnesota Multiphasic Personality Inventory (MMPI) [13], the MMPI-2 [14], and the Millon Clinical Multiaxial Inventory-III (MCMI-III) [15] evaluate personality traits and psychopathology, providing insights into coping mechanisms and symptomatology [35]. The Vocational Preference Inventory (VPI) [34] facilitates the identification of personality styles according to Millon’s framework [8]. Therapeutic assignment employs various instruments, such as the Therapeutic Reactance Scale (TRS) [17] for assessing resistance levels, the STS-Clinician Rating Form (STS-CRF) [18,19] for judgment-based profiling, and the Therapy Process Rating Scale (TPRS) [20] for session-coding [3,27]. Frequently utilized instruments comprise the Adult Attachment Interview (AAI) [21], the Beck Depression Inventory-II (BDI-II) [22], the Symptom Checklist-90-R (SCL-90-R) [23], the State-Trait Anxiety Inventory (STAI) [24], and the Dysfunctional Attitude Scale (DAS) [25]. Numerous tools are incorporated within multimodal frameworks, including the BASIC I.D. profile (behavior, affect, sensation, imagery, cognition, interpersonal relationships, drugs) [10,26,45].

RQ2: How Are Client Profiles Integrated Into Decision-Making Models for Selecting Appropriate Treatment Options?

Client profiling is often included into psychotherapy planning, utilizing models that vary from standardized instruments to dynamic feedback-informed approaches [28]. These models evaluate characteristics including readiness for change, interpersonal style, coping strategies, and responsiveness to different interventions. Prescriptive psychotherapy aligns treatment components with traits including rigidity, dysregulation, and resistance, which has specific implications for therapist behavior and therapeutic stance [28].

Various frameworks facilitate clinical decision-making. Empirical models like STS [3,5] provide structured algorithms that inform treatment-planning by considering resistance, coping mechanisms, symptom patterns, and functional status. These models frequently rely on treatment protocols categorized as empirically supported by the American Psychological Association’s Division 12 Task Force (https://div12.org/), which may reinforce preference for structured, manualized interventions. The predictive validity of these models surpasses that of unguided clinician judgment [27,29,41]. Furthermore, statistical approaches and algorithms in decision-making use machine learning or Bayesian modeling to incorporate client profiles and predict treatment response, intervention type, or therapist assignment based on baseline clinical characteristics or dynamic data (e.g., Ecological Momentary Assessment) [30-32]. These data-driven methods go beyond prescriptive frameworks by incorporating dynamic and individualized client information into structured decision-making processes.

Resistance has been thoroughly examined in the literature [5,9]. Clients with low resistance generally benefit from directive and structured techniques, whereas those with high resistance tend to respond more favorably to less controlling, client-centered approaches [5,9]. Motivational profiling is implemented through models such as the transtheoretical stages of change [33], which encompasses five distinct stages: precontemplation (no awareness of need for change), contemplation (awareness but ambivalence), preparation (commitment to change with initial steps), action (active behavior modification), and maintenance (sustained behavior change). These stages help clinicians select appropriate interventions for clients’ specific readiness levels and optimize the timing and pacing of change-oriented techniques [6,45]. Stage-based models align interventions with readiness stages, thereby optimizing the timing and pacing of change-oriented techniques [33].

Integrative profiling methods, such as adaptive counseling and therapy (ACT) [34], represent systematic approaches that continuously adjust therapeutic interventions based on the ongoing assessment of client characteristics, treatment response patterns, and emerging needs. These models emphasize flexibility in technique selection while maintaining structured decision-making processes. This allows therapists to modify their approach based on real-time feedback and indicators of client progress, which aim to enhance flexibility and structure. While ACT focuses on customizing techniques informed by continuous feedback, Arnold Lazarus’s multimodal therapy [26] takes a different approach by evaluating seven key dimensions of a client’s experience: behavior, affect, sensation, imagery, cognition, interpersonal relationships, and biological factors (BASIC I.D.) to facilitate theoretically integrated planning [26].

Feedback-informed care depends on outcome-monitoring and alliance-tracking to enhance alignment and avert deterioration [30,35]. Outcome-informed approaches utilize standardized measures to monitor client progress, facilitating real-time adjustments that enhance treatment fit and decrease dropout risk [30,35].

Trait-based profiling is also included in psychotherapy planning. Internalizing and externalizing coping styles are commonly utilized to distinguish intervention strategies [5]. Millon’s evolutionary model integrates evolutionary principles with personality theory. It categorizes individuals into distinct personality typologies based on three fundamental polarities: pleasure-pain (motivation), self-other (interpersonal focus), and active-passive (behavioral adaptation). Examples include the dependent type (other-focused, passive), the antisocial type (self-focused, active), and the avoidant type (pain-oriented, passive). These typologies inform therapeutic assignment by matching intervention strategies to personality structure and motivational dynamics categorizes personality traits and motivational dynamics into typologies that facilitate multimodal sequencing [8,36]. Selective and adaptive indication models enhance this approach by incorporating baseline predictors alongside contextual factors, including accessibility and client preferences, and modifying the treatment plan in response to initial outcome data [2,7,31]. Selective models utilize pre-treatment characteristics such as resistance and severity, whereas adaptive models permit real-time adjustments informed by session data [6,7,31].

The persistence of informal clinician-based decision-making is characterized by its idiosyncratic nature, which restricts replicability [4,39,48]. Some studies have emphasized the importance of experiential insights derived from client behavior and relational cues [36,37]. Mechanism-focused profiling highlights the importance of aligning intervention techniques with psychological processes such as avoidance or dysregulation [37,38]. Problem-centered and modular therapies have utilized profiling in a pragmatic manner, integrating interventions from various models according to client needs [39]. Mechanism-based models emphasize the fundamental psychological processes instead of diagnostic categories, thereby endorsing transdiagnostic treatment approaches [37,38]. Modular frameworks enable therapists to choose and arrange interventions according to specific case formulations [39].

Several studies have examined bidirectional models, indicating that therapist variables, including interpersonal style or preference, should be evaluated in conjunction with client characteristics. This preliminary research has suggested potential advantages for alliance development [30,42].

RQ3: What Client Variables Are Considered in Psychotherapy Assignment?

Multiple factors influence treatment alignment. Functional impairment and symptom severity have guided the design and focus of interventions, with greater dysfunction requiring more directive strategies [5]. Coping style serves as a crucial predictor. Internalizing clients generally respond favorably to insight-driven methods, while externalizers are more inclined to gain from structured, skills-based approaches [41].

Psychological mindedness affects the suitability of therapeutic modalities: Clients with introspective capacity, verbal-analytical skills, and logical-discursive reasoning abilities are more likely to engage effectively with insight-oriented therapies. Those without these cognitive capacities may benefit more from behavioral or experiential approaches [28]. Motivation and readiness for change, as defined by frameworks like the transtheoretical model, are essential in influencing both intensity and pacing [33].

Millon’s framework synthesizes personality traits, motivational systems, and clinical syndromes to inform treatment across diverse modalities [8]. This framework emphasizes structural and adaptive variables, offering a multiaxial classification for sequential and multimodal planning [8].

Cultural, relational, and preference-based variables were increasingly acknowledged. Clients’ beliefs, expectations concerning modalities, and relational needs significantly influence engagement and retention. The amalgamation of these factors has yielded positive outcomes [43,44]. Cultural background plays a crucial role in shaping expectations and communication styles. Culturally responsive strategies have been shown to reduce dropout rates and enhance partnerships, especially within minoritized groups [42]. Some contributions have supported incorporating therapist preferences into the matching process to improve relational fit [30,42].

Discussion

In this systematic review we examined methods, models, and assessment tools used to align clients with psychotherapeutic approaches, focusing on evidence-based strategies for personalized therapeutic assignment. We asked: (RQ1) What methods and assessment tools are utilized to align clients with psychotherapy? (RQ2) How are client profiles integrated into decision-making models for selecting appropriate treatment options? (RQ3) What client variables are considered in psychotherapy assignment? The review highlights a shift in the literature from intuition-based practices to evidence-informed therapeutic assignments. However, implementation gaps still remain. Our findings across the three research questions suggest that there have been advancements in assessment sophistication, but there are still ongoing barriers to clinical translation.

RQ1: Methods and Assessment Tools to Align Clients With Psychotherapy

Considerable advancements have been achieved in the conceptual framework of therapeutic assignment; nonetheless, notable constraints persist in its practical implementation. The discipline must advance beyond disorder-specific personalization to create tools and frameworks that are empirically robust and clinically applicable across diverse contexts. Addressing these challenges is important for enhancing treatment outcomes, minimizing trial-and-error approaches, and promoting a more inclusive and responsive mental health system.

The results of this review highlight the increasing empirical evidence that supports the alignment of psychotherapeutic methods with individual client characteristics through the use of structured assessment tools and decision-making models. Treatment assignment has traditionally depended on diagnosis and therapist preference. However, we observed a growing trend towards multidimensional profiling that emphasizes factors such as coping style, resistance, motivation, and psychological mindedness. Instruments like the MMPI-2 [14], MCMI-III [15], TRS [17], and STS-CRF [18,19] have demonstrated efficacy in assessing these dimensions and guiding clinical decision-making. The STS model [5] is distinguished among validated methods for integrating structured clinical judgment with algorithmic therapeutic assignment prescriptions.

RQ2: Client Profiling and Decision-Making Models

Despite the conceptual maturity of the reviewed tools, their clinical implementation needs further experimental investigation. Usage has been frequently confined to research environments or contexts with sufficient resources, with personalization initiatives mainly concentrating on applications related to specific disorders, especially depression and anxiety. This trend limits the generalizability of matching strategies, particularly in instances of comorbid or complex presentations. Trait-based models [3,8,24] provide guidance for treatment according to personality structure or motivational readiness. However, their scalability and practical integration into clinical workflows still needs further development. Adaptive models, including outcome-informed therapy, provide flexible frameworks that integrate real-time feedback and modify treatment based on changing client data, reflecting broader trends in patient-centered care and precision medicine.

RQ3: Relevant Client Variables

Our review identified several key client variables that are consistently associated with therapeutic assignment decisions. These variables include personality factors, such as coping style, resistance levels, and psychological mindedness; symptom profiles, including severity, complexity, and comorbidity patterns; cultural background and expectations; and motivation levels for change. A comparison of our findings with those of other systematic reviews in the therapeutic assignment literature reveals convergent results regarding the importance of coping style and resistance levels. However, our review uniquely emphasizes the significance of cultural factors and therapist-client compatibility. Previous reviews by Castonguay & Beutler [2] and Nye and colleagues [7] similarly identified personality factors and symptom severity as primary variables. Nevertheless, our findings extend these by highlighting the critical role of cultural responsiveness and the limitations of Western-centric assessment tools in diverse populations.

Many profiling systems appeared to inadequately represent sociocultural, relational, and contextual factors, despite their acknowledged significance [6,42,48]. Research has highlighted the significance of culture, expectations, and therapist-client compatibility. However, these factors have been infrequently incorporated into standardized assessment or decision-making protocols [35,42,43]. Consequently, informal clinical judgment has frequently prevailed in decision-making, supplanting structured methodologies with subjective assessments [4,6,28]. The dependence on intuition, although occasionally beneficial in clinical settings, results in variability and restricts reproducibility.

The research has demonstrated a trend towards increasingly structured methodologies in decision-making models. The STS framework [5] is consistently validated and provides empirically based criteria for guiding therapist behavior, modality selection, and session structure [10,27,47]. These models frequently rely on empirically validated treatments [5,39,42], thereby enhancing their credibility; however, this may favor structured, manualized approaches, especially cognitive-behavioral methods, while neglecting experiential, relational, or culturally tailored interventions [26,33,34].

The adaptability of theoretical orientations appeared relatively underexplored in the existing literature. Integrative and eclectic models were frequently regarded as superior for personalization. However, comparative studies that evaluated their flexibility across various client profiles were lacking. Cognitive behavioral therapy [40] provides customizable modular components, although clinical practice frequently adheres to established treatment protocols [5,26]. The apparent contradiction between the theoretical flexibility of CBT and its practical rigidity reflects institutional and training factors that favor adherence to protocols over individualized adaptations [37,39]. In contrast, psychodynamic and experiential therapies may provide greater relational flexibility, yet they often depend on clinical intuition instead of systematic matching procedures [6,43,46]. The lack of comparative evidence reinforces conventional approaches and hinders innovation in the creation of adaptive therapy models [7,34,37].

Challenges, Limitations, and Risk of Bias

This review was limited due to heterogeneity of study designs and our narrative synthesis approach, limiting generalizability and replicability. Many studies focused on Western clinical populations, reducing cross-cultural applicability. To mitigate bias, independent screening by three reviewers was used, but formal risk-of-bias assessment tools were not systematically applied.

Substantial challenges remain. The emphasis on disorder-specific personalization in various initiatives has limited the applicability of existing matching models which are inadequate for clients exhibiting complex, comorbid, or subthreshold symptomatology. Structured assessment tools are infrequently incorporated into standard clinical practice owing to resource limitations, insufficient training, and inadequate integration with electronic health systems [42]. The focus on randomized controlled trial standards for assessing treatment efficacy introduces a systemic bias that prioritizes manualized and symptom-focused interventions, leading to the underrepresentation of culturally sensitive or relationally attuned approaches [31]. This preference is reinforced by the classification systems used by professional organizations, such as the American Psychological Association’s (APA) Division 12 (https://div12.org/), which prioritize manualized treatments with randomized controlled trial support (i.e., treatments validated through studies that randomly assign participants to experimental or control conditions to ensure an objective evaluation of efficacy) [40].

Recent methodologies have sought to integrate client preferences and cultural values into treatment planning. However, these variables frequently lacked the operational precision characteristic of psychological traits [42]. The absence of methodological rigor in these domains interferes with the advancement of inclusive models and has perpetuated the dominance of restricted frameworks [43]. The ethical and clinical necessity to broaden therapeutic assignment beyond mere diagnosis and symptom severity is increasingly being recognized [1].

This systematic review has several key strengths, including a comprehensive search strategy across multiple databases, a rigorous methodology with independent reviewer screening, and a novel synthesis of therapeutic assignment approaches across diverse theoretical orientations. This review is the first to comprehensively map assessment tools and decision-making models in therapeutic assignment, offering valuable insights for clinical practice and research. The clinical implications are substantial: personalized therapeutic assignment can significantly improve treatment outcomes, reduce dropout rates, and enhance therapeutic efficiency. Healthcare organizations should invest in training programs for systematic assessment protocols and develop infrastructure that supports dynamic treatment planning.

Future research must focus on creating scalable and inclusive models that incorporate psychological, relational, and contextual factors. Comparative frameworks are still needed for evaluating the adaptability of various theoretical orientations to specific client characteristics. It is important to prioritize ecologically valid models that accurately represent the realities of diverse clinical populations and treatment settings. Methodologies that address complexity in client presentations and therapeutic processes are crucial for advancing personalized psychotherapy as an effective and equitable practice.

## Conclusions

This systematic review analyzes the evolution of therapeutic assignment strategies, shifting the focus from conventional, diagnosis-based models to more sophisticated approaches that consider multidimensional client profiles. An analysis of 47 studies reveals that coping style, resilience, motivation, and psychological insight are recognized as essential factors affecting the efficacy of therapeutic interventions. The STS model provides evidence-based guidelines for aligning therapeutic strategies with the unique needs of clients, but barriers to implementation persist. Notable limitations include a bias toward structured treatments, insufficient consideration of cultural factors, and gaps in adaptation where general assessment tools are repurposed rather than being designed specifically for therapeutic assignment.

The implementation of personalized approaches in clinical practice is constrained by resource limitations, insufficient training, and systemic biases favoring manualized interventions. This review emphasizes the need for transdiagnostic and culturally inclusive models, scalable assessment tools, and comparative analyses across theoretical orientations. Priority areas include developing implementation frameworks that bridge the gap between research and practice, creating technology-supported platforms for dynamic treatment planning, and establishing training programs for systematic assessment protocols. Comparative analyses of various theoretical orientations concerning client profiles are also crucial because aligning therapy with individual characteristics may enhance the efficacy and equity of mental healthcare.
